# RBOH-mediated ROS production facilitates lateral root emergence in *Arabidopsis*

**DOI:** 10.1242/dev.136465

**Published:** 2016-09-15

**Authors:** Beata Orman-Ligeza, Boris Parizot, Riet de Rycke, Ana Fernandez, Ellie Himschoot, Frank Van Breusegem, Malcolm J. Bennett, Claire Périlleux, Tom Beeckman, Xavier Draye

**Affiliations:** 1 Université Catholique de Louvain, Earth and Life Institute, Louvain-la-Neuve B-1348, Belgium; 2 Department of Plant Biotechnology and Bioinformatics, Ghent University, Ghent B-9052, Belgium; 3 Department of Plant Systems Biology, VIB, Ghent B-9052, Belgium; 4 Centre for Plant Integrative Biology, School of Biosciences, University of Nottingham, Sutton Bonington LE12 5RD, UK; 5 PhytoSYSTEMS, Laboratory of Plant Physiology, University of Liège, Sart Tilman Campus, 4 Chemin de la Vallée, Liège B-4000, Belgium

**Keywords:** Lateral root emergence, Reactive oxygen species, Auxin, Respiratory burst oxidase homologs, Auxin-mediated cell wall remodeling

## Abstract

Lateral root (LR) emergence represents a highly coordinated process in which the plant hormone auxin plays a central role. Reactive oxygen species (ROS) have been proposed to function as important signals during auxin-regulated LR formation; however, their mode of action is poorly understood. Here, we report that *Arabidopsis* roots exposed to ROS show increased LR numbers due to the activation of LR pre-branch sites and LR primordia (LRP). Strikingly, ROS treatment can also restore LR formation in *pCASP1:shy2-2* and *aux1 lax3* mutant lines in which auxin-mediated cell wall accommodation and remodeling in cells overlying the sites of LR formation is disrupted. Specifically, ROS are deposited in the apoplast of these cells during LR emergence, following a spatiotemporal pattern that overlaps the combined expression domains of extracellular ROS donors of the RESPIRATORY BURST OXIDASE HOMOLOGS (RBOH). We also show that disrupting (or enhancing) expression of RBOH in LRP and/or overlying root tissues decelerates (or accelerates) the development and emergence of LRs. We conclude that RBOH-mediated ROS production facilitates LR outgrowth by promoting cell wall remodeling of overlying parental tissues.

## Abstract (precis)

**Summary:** Reactive oxygen species promote cell wall remodeling of cells overlying the sites of lateral root formation, thereby contributing to lateral root emergence in *Arabidopsis*.

## INTRODUCTION

Root branching plays a crucial role enhancing the ability of the root system to explore and take up water and nutrients from the soil environment. In the model plant *Arabidopsis*, lateral roots (LRs) are derived from pairs of xylem pole pericycle cells located deep within the primary root (Dubrovsky et al., 2006; Himanen et al., 2002; Jansen et al., 2013; Malamy and Benfey, 1997). The hormone auxin plays a key role during early developmental stages of LRP (Casimiro et al., 2001). Increased auxin levels mediated by auxin influx and efflux transporters (Benkova et al., 2003; Marchant et al., 2002; Marhavy et al., 2013) are perceived by TIR1 and AFB receptors and trigger degradation of different AUX/IAA repressors of auxin response transcription factors (ARFs), releasing the expression of auxin-responsive genes (De Smet, 2011; Lavenus et al., 2013).

Early auxin-response modules controlling LRP formation, namely *ARF7* and *ARF19* (Okushima et al., 2007), *SLR* (also known as *IAA14*) (Fukaki et al., 2002), *IAA28* (Rogg et al., 2001) and *SHY2* (*IAA3*) (Goh et al., 2012; Hosmani et al., 2013; Tian and Reed, 1999; Vermeer et al., 2014), operate within the LRP and in the tissues of the parental root that overlie the LRP to coordinate its initiation and emergence (Swarup et al., 2008). It is now clear that auxin-mediated modifications of cell wall properties represent an essential step during LR development. In the endodermis, the *SHY2* signaling module triggers changes in cell volume and wall properties termed ‘spatial accommodation’, thereby facilitating the passage of LRP (Vermeer et al., 2014). In the cortex and the epidermal cells overlying the expanding LRP, cell wall remodeling enzymes are induced to facilitate LRP emergence (Gonzalez-Carranza et al., 2007; Lewis et al., 2013; Neuteboom et al., 1999; Swarup et al., 2008). The activity of the auxin influx carrier LIKE AUX1 3 (LAX3) localizes the auxin-induced expression of these cell wall remodeling genes that degrade the pectin-rich middle lamellae. In agreement with this, LRP emergence through the cortex and epidermis is hampered in *lax3* mutants (Swarup et al., 2008) and defects in genes involved in cell wall formation increase the rate of LRP emergence, as shown recently with mutants with impaired cell wall biosynthesis (Roycewicz and Malamy, 2014) and abscission (Kumpf et al., 2013).

In addition to hormones like auxin, there is compelling evidence that ROS also function as signaling molecules during plant development, as shown for several signal transduction pathways (D'Haeze et al., 2003; Ishibashi et al., 2012; Joo et al., 2001; Mori et al., 2001) and developmental events such as xylem differentiation (Ros Barcelo, 2005), root gravitropism (Joo et al., 2001), adventitious root formation (Liao et al., 2012) and root-to-shoot coordination (Passaia et al., 2013). Recent evidence also suggests that ROS act during LR formation (Correa-Aragunde et al., 2013; Li and Jia, 2013; Manzano et al., 2014) in relation to auxin response (Correa-Aragunde et al., 2013; Ma et al., 2014), but the mechanistic basis of this crosstalk remains unclear. Among ROS, O_2_^−^ and H_2_O_2_ were shown to be involved in cell wall modifications during several plant developmental processes (Carol et al., 2005; Foreman et al., 2003; Monshausen et al., 2007; Ros Barcelo, 2005). The production of ROS in extracellular spaces depends on several classes of enzymes, including respiratory burst oxidase homologs (RBOH) and class III peroxidases (Sagi and Fluhr, 2006; Shapiguzov et al., 2012). Interestingly, the latter enzymes appear to regulate root branching in an auxin-independent manner (Manzano et al., 2014). To date, it has not been determined whether RBOH are involved in the auxin-mediated signaling leading to cell wall remodeling during LR formation.

In this study, we exploit gene expression datasets to highlight the existence of interplay between ROS and auxin signaling pathways during early steps of LR formation and we show that exogenous application of ROS can rescue LR-less mutants that are defective in auxin signaling in tissues overlying new LRP. Using high-resolution imaging, we reveal that ROS accumulate in the middle lamella of these cells. In addition, spatial expression analysis of several auxin-inducible RBOH genes during LR formation suggests that their activity cause the production of extracellular ROS during this developmental process. Finally, functional studies employing RBOH mutants and the tissue-specific overexpression of *RBOHD* validate the importance of this gene family in facilitating LRP emergence.

## RESULTS

### An interplay between auxin and ROS signaling during LR formation

We initially analyzed datasets from published microarray experiments (Affymetrix ATH1 arrays) that relate to auxin-mediated LR formation or ROS responses. The experiments involving auxin employed the LR inducible system (LRIS; Himanen et al., 2002; Jansen et al., 2013) and allowed us to pinpoint genes potentially involved in rapid transcriptional response to auxin and most likely involved in LR formation. In the LRIS system, seedlings are grown for 3 days on the auxin transport inhibitor 1-N-naphthylphthalamic acid (NPA) and then treated for 2 h with synthetic auxin-related signaling molecules 1-naphthaleneacetic acid (NAA) or naxillin to trigger synchronous LR formation in root pericycle cells (De Rybel et al., 2012; Vanneste et al., 2005). For experiments relating to ROS, 5-day-old seedlings were treated for 1 h with 20 mM H_2_O_2_ (Davletova et al., 2005) or 2-week-old seedlings were sprayed for 3 h with 20 mM H_2_O_2_ (Ng et al., 2013). A list of 108 overlapping genes (out of 489 genes from the two auxin experiments and 414 genes from at least one of the two H_2_O_2_ experiments) were selected employing cut-offs of an absolute fold change ≥2 and a *P*-value ≤0.05 (Table S1). Of these 108 genes, 90 genes were simultaneously induced in auxin and H_2_O_2_ datasets but only two were repressed in both. Furthermore, 13 of the genes were induced during LR formation and were found to relate to redox activity, and 24 were linked to stress response, suggesting that fine-regulation of redox balance is necessary during auxin-mediated LR formation. Consistent with this model, exogenous auxin application increased ROS levels in root tissues (Fig. S1A,B). Hence, our results suggest a link between ROS and auxin-mediated LR formation.

### ROS application activates LR pre-branch sites

**Fig. 1.**
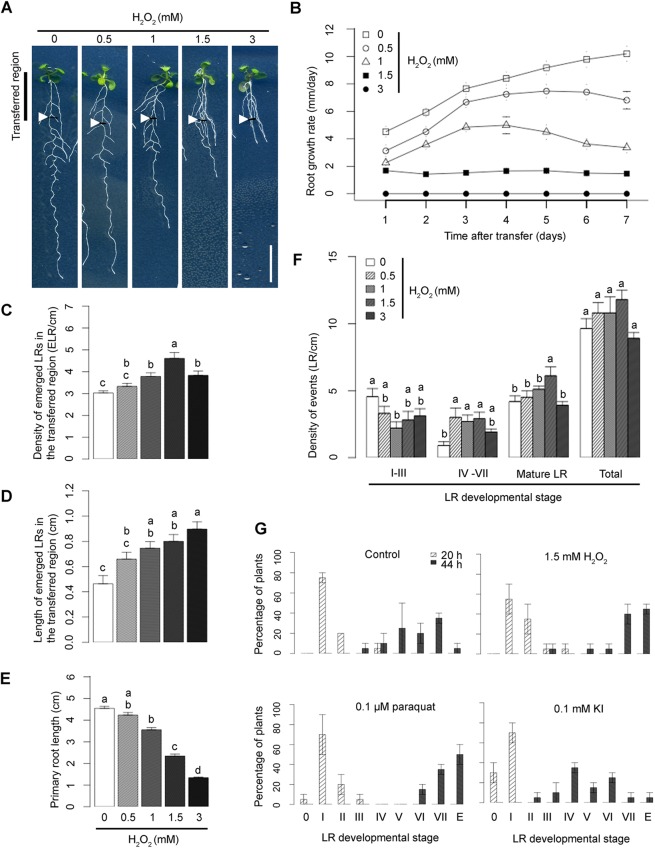
**The** **effect of ROS on root development.** (A) Morphology of Col-0 grown in control conditions and upon treatment with increasing H_2_O_2_ concentrations. Five-day-old seedlings (transferred region) were exposed to H_2_O_2_ for 7 days. White arrowheads indicate the root tip region at the moment of transfer. Scale bar: 1 cm. (B) Primary root (PR) growth rates upon treatment with increasing H_2_O_2_ concentrations. Five-day-old seedlings were transferred onto media supplemented with increasing concentrations of H_2_O_2_. The root tips of the seedlings were marked each day. After 7 days, the distances between each mark were measured and the average root growth for each time point (technical replicates, *n*=15 per sample) is shown in the graph. (C,D) Average emerged LR density (C) and LR length (D) in transferred region after 7 days of H_2_O_2_ treatment (in three biological replicates, *n*=30). Owing to a strong effect of H_2_O_2_ treatment on primary root growth rates, LR density and length were calculated only for the transferred regions of the root. (E) Average PR length after 7 days of H_2_O_2_ treatment (in three biological replicates, *n*=30). (F) Effect of ROS on LRP density after 2 days of different concentrations of H_2_O_2_. (C-F) The difference between groups denoted by different lowercase letters is statistically significant (*P*<0.005 according to Tukey's HSD test after ANOVA). (G) Effect of ROS and ROS scavengers on LR emergence phenotype. Five-day-old seedlings were transferred onto media supplemented with various compounds, as indicated above each graph and gravistimulated by turning the plates 90° to achieve synchronization of LR formation. LRP stages according to Dubrovsky et al. (2006), Himanen et al. (2002), Jansen et al. (2013) and Malamy and Benfey (1997), starting from stage I to an emerged LR (E), are shown on *x*-axis. Data points represent mean±c.i. (in two biological replicates, *n*=20).

Seedlings exposed to H_2_O_2_ have been previously reported to exhibit an increase in LR number compared with control seedlings (Manzano et al., 2014). We validated this by exposing root segments to H_2_O_2_, which increased LR density and length in the exposed segments, whereas root growth rate decreased in a dose-dependent manner after onset of the treatment (Fig. 1A-E). The effect of H_2_O_2_ on primary root growth is unlikely to be caused by toxicity as it was reversed completely (for 1 mM H_2_O_2_) or partially (1.5 mM H_2_O_2_) within 2 days of transfer back on control medium (Fig. S1C). A permanent arrest of the primary root growth was only observed at 3 mM of H_2_O_2_.

To investigate further how H_2_O_2_ application impacts LR development, 5-day-old seedlings were exposed to H_2_O_2_ for 2 days (Fig. 1F). Upon H_2_O_2_ treatment, the number of emerged LRs increased, whereas the number of early stage LR primordia decreased. *Arabidopsis* seedlings produce an excess of LR pre-branch sites, but only a subset will be used for LR production (Van Norman et al., 2014). We used a modified LR inducible system (Himanen et al., 2002; Jansen et al., 2013) to explore the possibility that H_2_O_2_ treatment promotes the developmental progression of LRs from these unused precursor sites rather than inducing *de novo* LR formation. LR formation was synchronized by germinating *pDR5:GUS* transgenic seedlings for 3 days in the presence of 10 μM NPA followed by transfer onto control media or media supplemented with H_2_O_2_ (1.5 mM), the ROS scavenger potassium iodide (KI; 0.01 mM), both H_2_O_2_ and KI (1.5 mM and 0.01 mM, respectively), or NAA (10 μM; positive control). Samples were collected at 6 h, 12 h and 18 h after transfer and histochemically stained for GUS activity. In control conditions and upon KI treatment, GUS-positive foci, representing LR founder cells and initiation sites, appeared within 12 h, whereas in 86% of seedlings grown in the presence of H_2_O_2_, GUS-positive foci were already observed within 6 h. (Fig. S2A,B). Interestingly, the latter appeared in similar locations compared with control conditions, unlike upon NAA treatment, where synchronous LR formation was induced equally along the root. Hence, our results indicate that ROS facilitates early developmental events leading to LRP formation but does not induce *de novo* LR initiation.

To uncover the effect of ROS on the kinetics of LR development, we employed the root bending assay (Fig. 1G), in which roots are given a 90° gravistimulus to synchronize LR initiation and emergence in the resulting root bend (Peret et al., 2012b) and LRP stages are counted 20 and 44 h after gravistimulation (hag) according to the methods of Malamy and Benfey (1997). In parallel to the plate rotation, seedlings were treated with H_2_O_2_ (1.5 mM), the O_2_^−^ donor methyl viologen dichloride hydrate (paraquat; 0.1 μM) or the ROS scavenger KI (0.1 mM). At 20 hag, control roots accumulated mainly stage I LRP. Seedlings treated with ROS donors exhibited a higher percentage of stage II and III in comparison with the control, whereas KI-treated seedlings showed a decrease in stage I LRP. At 44 hag, control plants accumulated mainly stage V, VI and VII LRP. Seedlings treated with ROS donors were more advanced than control seedlings and showed stage VII LRP and emerged LRs, whereas KI-treated seedlings showed a delay in LR emergence in which stages IV to VII were noted.

### ROS treatment bypasses the requirement for auxin influx carrier activity during LR initiation and emergence

**Fig. 2.**
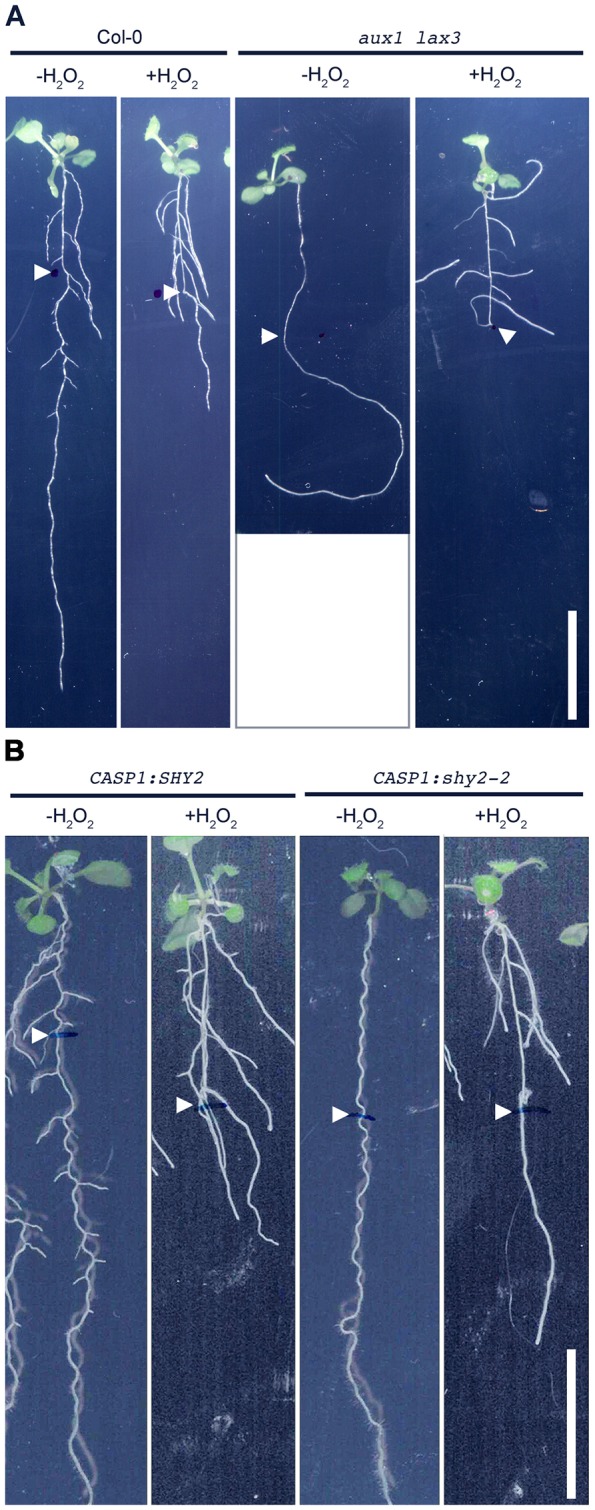
**The effect of ROS on the LR phenotype of auxin mutants.** (A) Effect of exogenous H_2_O_2_ on LR formation in Col-0 (control) and *aux1 lax3* background. (B) Effect of exogenous H_2_O_2_ on LR formation in *pCASP1:SHY2* (control) and in *pCASP1:shy2-2* gain-of-function background. Five-day-old seedlings were exposed to H_2_O_2_ (1.5 mM) for 7 days. White arrowheads indicate the root tip region at the moment of transfer. Scale bars: 1 cm.

To assess the capacity of H_2_O_2_ to promote LR formation, we investigated whether ROS treatment could rescue mutations disrupting early steps of LR development. *AUX1* and *LAX3* encode members of a family of auxin influx carriers that are required for LR initiation and emergence, respectively (Lavenus et al., 2013). The combined loss of both genes results in a lateral rootless mutant phenotype (Fig. 2A; Swarup et al., 2008). Strikingly, H_2_O_2_ treatment (1.5 mM) of 5-day-old seedlings of the double *aux1 lax3* mutant for 7 days resulted in the appearance of emerged lateral roots (Fig. 2A). We found that LR densities were 3.7±0.4 for *aux1 lax3* seedlings (*n*=36) exposed to H_2_O_2_ and 3.0±0.2 (*n*=35) and 5.1±0.5 (*n*=37), respectively, for wild-type seedlings in control conditions and exposed to H_2_O_2_ (LR/cm, mean±c.i.). Next, evaluating sensitivity to H_2_O_2_ with respect to primary root growth showed that *aux1 lax3* is equally sensitive to H_2_O_2_ as the control wild-type seedlings. In control conditions, primary root growth rate of 5-day-old wild-type plants transferred to a new control medium for 3 days is similar to *aux1 lax3* (7.2±1.3 and 7.32±1.08, respectively; mm/day, *n*=15). Similarly, upon treatment with 1.5 mM H_2_O_2_ for 3 days, root growth decreased equally in wild type and in *aux1 lax3* genetic backgrounds (1.35± 0.64 and 1.77±0.52, respectively; mm/day, *n*=15). However, the *aux1 lax3* root gravitropic defect was not rescued (Fig. 2A). Our results suggest that H_2_O_2_ treatment does not influence shootward auxin transport driven by AUX1, which is required for gravitropism, but rather overcomes the absence of the auxin gradient that has been shown to induce the expression of cell wall remodeling genes in the overlying cell layers, which is needed for LR emergence (Swarup et al., 2008).

Auxin efflux carrier activity is also important for LR development (Benkova et al., 2003; Casimiro et al., 2001). The *gnom^R5^* mutation (in an ARF GDP/GTP exchange factor involved in polar localization of the auxin efflux regulator PIN1) represents a weak allele and produces an embryonic root devoid of emerged LRs (Geldner et al., 2004). H_2_O_2_ treatment of *gnom^R5^* seedlings did not overcome the LR phenotype (Fig. S2C) and no massive proliferation of pericycle cells was observed after tissue clearing, indicating that its promoting effect is at least in part dependent on correct GNOM- and PIN1-mediated auxin transport.

To validate our genetic results, we also tested the effects of H_2_O_2_ when co-treating roots with inhibitors of auxin influx [1-naphthoxyacetic acid (1-NOA; 10 μM)] and efflux [NPA (1 μM) and 2,3,5-triiodobenzoic acid (TIBA; 10 μM)], which are known to disrupt early steps of LR formation (Casimiro et al., 2001; Peret et al., 2013). We observed that H_2_O_2_ treatment bypassed only the inhibitory effects of 1-NOA on LR formation (Fig. S2D,E). We conclude that ROS can bypass impaired influx-dependent auxin accumulation but not defects in auxin efflux carrier transport, as corroborated by the *gnom^R5^* data.

### ROS contributes to cell wall remodeling during LRP development

The auxin influx carrier LAX3 facilitates the accumulation of auxin in cortical and epidermal cells directly overlying new LR primordia, resulting in the induction of cell wall remodeling enzymes to facilitate organ emergence (Swarup et al., 2008). As H_2_O_2_ treatment can overcome impaired cell wall remodeling in cortex and epidermis in the *lax3* background, we tested whether this observation holds also true for plants with disrupted auxin-dependent endodermal cell wall remodeling. Transgenic lines expressing *pCASP1:shy2-2* are specifically disrupted in their endodermal auxin response, resulting in an LR-less phenotype (Goh et al., 2012; Hosmani et al., 2013; Vermeer et al., 2014). Strikingly, treatment with 1.5 mM H_2_O_2_ rescued LR development in the *pCASP1:shy2-2* gain-of-function mutants (Fig. 2B). By contrast, neither LRP nor LRs could be induced in mutants in which LR formation is compromised due to defects in pericycle auxin signaling, such as *iaa28* (Rogg et al., 2001), *arf7 arf19* (Okushima et al., 2007) and *slr* (Fukaki et al., 2002), suggesting that H_2_O_2_ plays a specific role during auxin-mediated wall remodeling in cells overlying new LR primordia (Fig. S3A).

Localized root cell wall remodeling has been reported to be associated with changes in extracellular pH (Bibikova et al., 1998; Monshausen et al., 2007; Vermeer et al., 2014), prompting us to investigate whether H_2_O_2_ modifies extracellular pH during LRP development. Using the apo-pHusion reporter line (Gjetting et al., 2012), we observed significant apoplast acidification in the parental ground tissue after 1 day of H_2_O_2_ treatment (Fig. S3B). We therefore hypothesize that exposure to H_2_O_2_ triggers wall acidification in cells overlying LR primordia to facilitate cell wall remodeling and organ emergence.

### ROS are detected in the middle lamellae of cells overlying developing LRs

Localization of ROS during LRP development has recently been reported employing a whole-mount staining assay in *Arabidopsis* (Manzano et al., 2014) and maize (Fig. S3C). We corroborated these observations at a cellular level of resolution using confocal microscopy combined with 2′-7′-dichlorodihydrofluorescein diacetate (DCFH-DA, 50 μM) to detect ROS (Aranda et al., 2013). Confocal imaging indicated strong DCFH-DA fluorescence surrounding cortex cells that overlay LRP (Fig. S3D), consistent with ROS playing a role during cell wall remodeling.

**Fig. 3.**
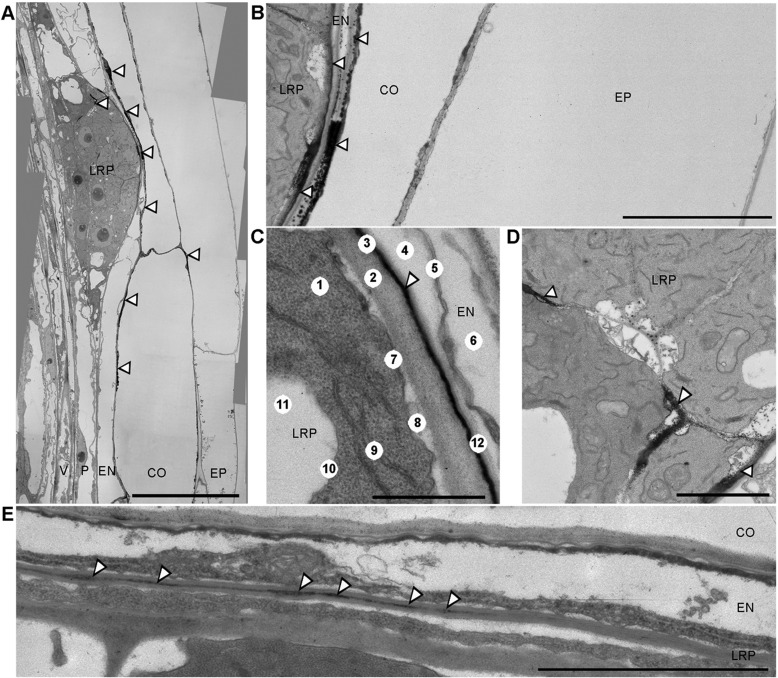
**Representative transmission electron microscopic images of *Arabidopsis* LRP treated with cerium chloride to visualize localization of H_2_O_2_ by black cerium depositions.** (A-D) H_2_O_2_ localization during LR emergence in outer cells (B), between LRP and endodermis (C), and between flanking cells inside the LRP (D). (E) H_2_O_2_ localization in LRP at stage II of development in middle lamellae between outer cells of LRP and endodermis, as the LRP is passing through endodermis. B and D are magnified views of A. CO, cortex; EN, endodermis; EP, epidermis; LRP, lateral root primordium; P, pericycle; V, vasculature. Numbers in C indicate (1) cytoplasm, (2) cell wall of outer LR cell, (3) middle lamella, (4) periplasmatic space, (5) remnants of endodermis protoplast, (6) vacuole, (7) plasma membrane, (8) periplasmatic space, (9) endoplasmic reticulum, (10) tonoplast, (11) vacuole and (12) cell wall of endodermis cell. Scale bars: 20 μm (A); 6 μm (B); 2 μm (C-E). Magnifications: 1200× (A); 4000× (B); 12,000× (C-E). White arrowheads point to the cerium depositions. *n*=15.

To resolve the subcellular localization of the most stable ROS species during LRP development, we employed transmission electron microscopy (TEM) to detect black cerium precipitates, which indicate the presence of H_2_O_2_. Our TEM approach detected H_2_O_2_ within the middle lamellae of cell walls, a pectin-based layer that cements the walls of adjacent cells together (Fig. 3; Table S2). H_2_O_2_ accumulation was observed in the middle lamellae of cortical and endodermal cells overlying new LRP. The fine layer of H_2_O_2_ covering the entire LRP clearly separated the LRP from parental tissues (Fig. 3B,C). In addition, cerium precipitates were detected inside LRP, particularly within the middle lamellae of cells at their flanks (Fig. 3D). Hence, H_2_O_2_ is deposited in the middle lamellae of cells in contact with, and also flanking, LRP during organ emergence.

### An auxin-inducible family of RBOH NADP oxidases produces extracellular ROS to facilitate LR development

**Fig. 4.**
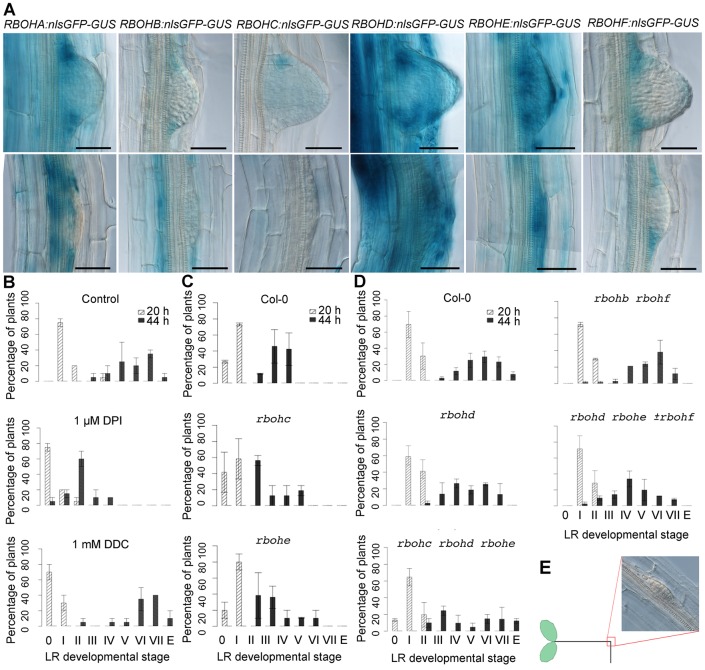
**Expression pattern of RBOH genes during LR development and RBOH-mediated effect on LR emergence phenotype.** (A) Promoter activities of RBOH genes during LRP development. Seven-day-old seedlings of each *pRBOH:nlsGFP:GUS* line, as indicated, were GUS stained. Scale bars: 50 μm. (B) Effect of the superoxide dismutases blocker diethyldithiocarbamate (DDC, 1 mM), and the RBOH inhibitor diphenyleneiodonium chloride (DPI, 1 μM) on LR emergence phenotype, starting from stage I to an emerged LR (E on *x*-axis). Data points represent mean±c.i. (in two biological replicates, *n*=20). (C,D) LR emergence phenotype in wild type and RBOH single and higher order mutants, as indicated. Data points represent mean±c.i. (in two biological replicates, *n*=20). (E) A synchronization of LRP initiation (20 h) and emergence (44 h) is achieved by gravistimulation and occurs at the bending site.

Given the importance of extracellular ROS deposition during LR development, we investigated the spatial expression of several RBOH genes known to contribute to ROS production. The *Arabidopsis* genome contains ten RBOH genes, named *RBOHA* to *RBOHJ* (accession numbers: RBOHA, At5g07390; RBOHB, At1g09090; RBOHC, At5g51060; RBOHD, At5g47910; RBOHE, At1g19230; RBOHF, At1g64060; RBOHG, At4g25090; RBOHH, At5g60010; RBOHI, At4g11230; and RBOHJ, At3g45810), expression of which in various organs has been related to different developmental processes (Boisson-Dernier et al., 2013; Foreman et al., 2003; Kwak et al., 2003; Lee et al., 2013; Muller et al., 2009; Torres et al., 2002). During LRP formation, the spatial expression patterns of GUS transgenes driven by various RBOH promoters largely overlap with H_2_O_2_ localization in the peripheral cells of the LRP (Fig. 4A). *RBOHE* was also strongly expressed in endodermis, cortex and epidermis cells overlying LRP (Fig. 4A; Fig. S4A). Interestingly, *RBOHA*, *RBOHC* and *RBOHE* were also expressed in the basal meristem (Fig. S4B), where LR priming occurs (De Smet et al., 2007) and expression of *RBOHE* is independent of AUX1 and LAX3 (Fig. S4C). Similarly, H_2_O_2_ treatment did not affect *AUX1* or *LAX3* promoter activities (Fig. S4D). Taken together, the expression pattern of RBOH genes inside the developing LRP and the overlying endodermis, cortex and epidermis cells are consistent with NADPH oxidase family members providing the extracellular ROS observed during LR development.

To overcome a potential genetic redundancy within RBOH family members, we employed treatments with the inhibitors of intra- and extracellular enzymes in parallel to the root bending assay. We used the RBOH inhibitor diphenyleneiodonium chloride (DPI, 1 μM) and diethyldithiocarbamate (DDC, 1 mM), which is known to affect the conversion of O_2_^−^ into H_2_O_2_ (Fig. 4B). Whereas control roots at 20 hag accumulated mainly stage I LRP, very few LRP were noticed in inhibitor-treated seedlings. At 44 hag, control plants accumulated mainly stage V, VI and VII LRP. Although no remarkable differences from the control were observed upon treatment with DDC, mostly stage II was detected in DPI-treated seedlings. To determine whether ROS produced by specific RBOH enzymes contribute to LR development, we analyzed root phenotypes of mutant lines lacking selected individual or combinations of RBOH genes. LR phenotyping of several RBOH mutants revealed a delay in the rate of organ emergence for selected lines (Fig. 4C,D). In particular, higher-order mutants lacking family members *RBOHE* and/or *RBOHD* were observed to have the strongest phenotype, consistent with both genes exhibiting the strongest and most widespread expression in overlying tissues (Fig. 4A) in the root bending assay (Fig. 4E). In summary, our genetic and pharmacological studies indicate that extracellular ROS donors contribute to LRP development.

**Fig. 5.**
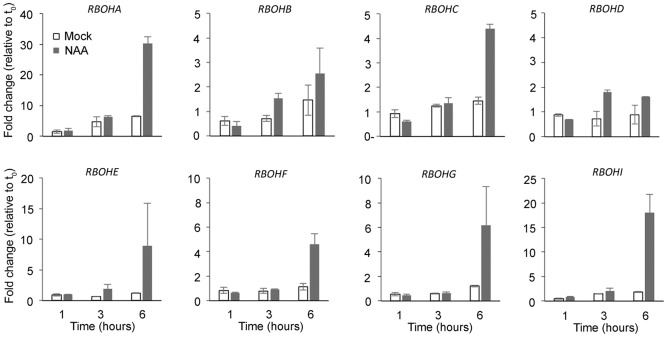
**Relative RBOH transcript levels in root tissue.** RBOH transcript levels were measured by qRT-PCR after 1, 3 and 6 h NAA treatment. The data are shown for two independent biological replicates±s.e.

We next investigated the possibility that expression of RBOH genes is auxin inducible. For this purpose, we employed qRT-PCR analysis and focused on RBOH transcript levels in root tissue of young seedlings. Upon treatment with NAA for a given duration, all RBOH transcripts detected in root tissue were upregulated by auxin already within 6 h of treatment (Fig. 5), in agreement with several published transcriptome datasets (Table S1). Hence, auxin was able to induce a strong upregulation of all members of the RBOH gene family expressed in roots.

### Tissue-specific overexpression of RBOH promotes LR emergence

In our experimental conditions, seedlings of the *35S:RBOHD* line showed many different developmental phenotypes making it impossible to distinguish between the effect of the constitutive expression on LR emergence from secondary effects on plant development (Fig. S4E), probably due to an overall increase in extracellular ROS levels (Mersmann et al., 2010). To determine which specific cell types were most sensitive to ROS accumulation during LR emergence, we targeted RBOH expression to selected root tissue(s) by crossing a homozygous *UAS:RBOHD* line with various GAL4-GFP enhancer trap lines. These included lines expressed in pericycle (J2661), endodermis and cortex (J3611), epidermis (J0634), simultaneously in LRP and overlying tissues (J0192) or in LRP alone (J1103).

**Fig. 6.**
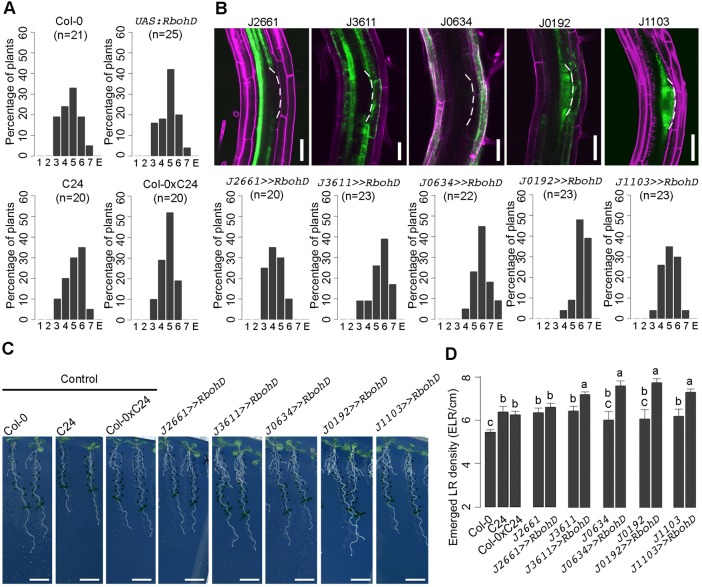
**The effect of tissue-specific overexpression of *RBOHD* on LR development.** (A) LR emergence phenotype of control lines. Five-day-old seedlings were transferred onto new media and gravistimulated by 90° to achieve synchronization of LR formation. LRP were grouped according to developmental stages at 44 h after the onset of gravistimulation. Data points represent mean±c.i. (technical replicates). E, emerged LR. (B) Expression pattern of GAL4 transactivation lines (upper panels) and LR emergence phenotype of *UAS:RBOHD* targeted to the corresponding GAL4 transactivation lines, as indicated. Scale bars: 50 μm. (C) Representative root phenotypes of 10-day-old seedlings. (D) Emerged LR number of control and tissue-specific transactivation lines of *UAS:RBOHD*. Data points represent mean±c.i. (technical replicates, *n*=20). The difference between groups denoted by different letters is statistically significant (*P*<0.001 according to Tukey's HSD test after ANOVA).

The phenotypic effect of targeted *RBOHD* overexpression on LR emergence was analyzed using the root bending assay (Peret et al., 2012a) and stages of synchronized LRP development were counted at 44 hag. The control Col-0, C24, *UAS:RBOHD* and Col-0×C24 seedlings accumulated mainly stage V LRP (Fig. 6A). The activation of *UAS:RBOHD* construct in the root pericycle and LRP alone had no effect on LR emergence compared with controls, where LRP accumulated mainly at stage V. By contrast, LR emergence was accelerated when *UAS:RBOHD* overexpression was targeted to the LRP and overlying tissues or only to the overlying root tissues, where LRP accumulated mainly at stage VI and VII (Fig. 6B). Similarly, when we observed the emerged LR density in 10-day-old seedlings, we observed an increased emerged LR density when *RBOHD* expression was transactivated in LRP and/or overlying root tissues (Fig. 6C,D). In summary, targeted *RBOHD* overexpression in LRP and/or overlying root tissues promotes organ emergence, in agreement with (sub)cellular distribution of H_2_O_2_ (Fig. 3).

## DISCUSSION

### ROS act downstream of auxin

Multiple auxin response modules are sequentially activated during successive developmental steps leading to the formation of LRs (Lavenus et al., 2013). We report here that auxin is able to induce expression of several RBOH genes and that changes in expression of ROS-related genes are associated with early steps of auxin-induced LR formation. This corroborates previous reports that demonstrated ROS production to occur downstream of auxin*-*mediated signal transduction pathways (Correa-Aragunde et al., 2013; Ivanchenko et al., 2013; Joo et al., 2001; Ma et al., 2014). In line with their potentially harmful effects, the production of ROS compounds in the apoplast is targeted to restricted spatial and temporal domains within plant organs (Bibikova et al., 1998; Monshausen et al., 2007; Vermeer et al., 2014). In response to unfavorable environmental conditions, such as salinity and drought, LR development is inhibited (De Smet et al., 2006; Duan et al., 2013). It is tempting to speculate that the activation of ROS scavenging machinery that probably occurs during exposure to abiotic stress (Caverzan et al., 2012) interferes with RBOH-mediated ROS production and/or removal from the apoplast, thereby affecting LR development.

Auxin signaling modules, which control LR development both in the LRP and in overlying tissues (Lavenus et al., 2013), are good candidates for the restriction of spatiotemporal ROS production to appropriate cell wall domains. Our additional observation that increased H_2_O_2_ levels (supplied externally or most likely by tissue-specific overexpression of *RBOHD*) accelerate the early steps of LR formation further suggests that the LRP and/or the overlying tissues are, at some point, receptive to a signal arising downstream of ROS. Taken together, we propose that auxin triggers RBOH-mediated ROS production where needed, thereby initiating the subsequent steps of LR formation.

### ROS action on cell wall remodeling

Auxin-regulated changes in wall properties of cells overlying LRP are indispensable for successful LR formation (Swarup et al., 2008; Vermeer et al., 2014). In this study, we demonstrated that ROS treatment can bypass the suppression of expression of genes involved in cell wall remodeling in *aux1 lax3* and *pCASP1:shy2-22* backgrounds*.* We also observed that the tissue zone in which H_2_O_2_ was recorded in the middle lamellae during LRP development largely corresponds to the expression patterns of several RBOH enzymes known to produce extracellular O^2−^ (Sagi and Fluhr, 2006). Given the relevance of peroxidases producing H_2_O_2_ from O^2−^ and their promoting effect on LR formation (Manzano et al., 2014), RBOH enzymes probably serve as O^2−^ donors for peroxidases during this developmental process in defined locations. However, we cannot exclude the possibility that RBOH and peroxidases are acting independently, as conversion of O^2−^ to H_2_O_2_ can also occur spontaneously, without any enzymatic support. Pharmacological inhibition of every RBOH enzyme severely impeded LRP development, suggesting that several RBOH enzymes are likely to be involved. Among the members of the RBOH gene family, the auxin-inducible *RBOHE* was expressed inside the LRP and in overlying cells of the endodermis, cortex and epidermis. These results support the hypothesis that extracellular ROS are directly involved in the modification and/or degradation of the middle lamellae in front of LRP.

### Role of ROS in overlying tissues in LR emergence

A major displacement in cell position occurs as the expanding LRP traverses the cortex and epidermis layers. In cortical and endodermal cells, LAX3 activity promotes auxin-dependent induction of cell wall remodeling enzymes such as SUBTILISIN-LIKE PROTEASE (AIR3), PECTATE LYASE (PLA2) and XYLOGLUCAN ENDOTRANSGLYCOSYLASE (XTR6) (Swarup et al., 2008). The degradation of the middle lamellae by ROS is likely to be a part of the machinery allowing slipping of the cell wall at the boundary between the outer layer cells of the LRP and the neighboring endodermis, cortex and epidermis cells as the LRP expands. Previous studies reported that ROS treatment increases LR number (Correa-Aragunde et al., 2013; Ma et al., 2014). However, we show here that exogenous ROS treatment does not induce the formation of *de novo* LR initiation sites, but rather promotes the developmental progression of the existing LRP and LR pre-branch sites, leading eventually to increased emerged LR numbers. Restoration of LR formation capacity by ROS treatment of *pCASP1:shy2-2* mutants further corroborates our hypothesis that ROS are mediating lateral root development through their action on cell wall mechanics because the incompetence of these mutants to form lateral roots is attributed to the lack of spatial accommodation. However, we cannot exclude the involvement of a downstream ROS signaling cascade in this process.

### RBOH-mediated ROS production promotes LR emergence

**Fig. 7.**
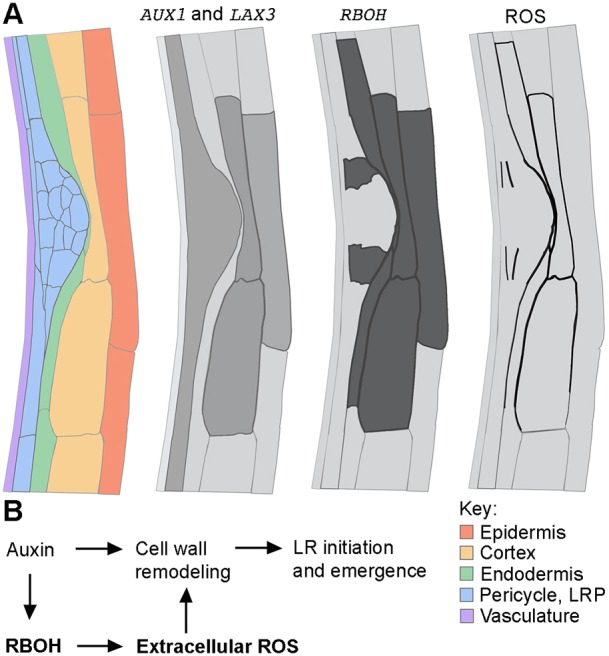
**Linking RBOH-mediated ROS production to the current understanding of auxin-mediated LR formation.** (A) The expression patterns of RBOH genes overlap with ROS localization and promoter activities of auxin influx carriers during LR emergence (longitudinal section). *AUX1* is expressed inside LR primordia and in the pericycle, whereas *LAX3* is expressed in the cortex and epidermal cells in front of emerging LR primordia (Swarup et al., 2008; Swarup and Peret, 2012). The promoters of RBOHs are active in peripheral cells of the LRP and in cells surrounding the emerging LRP. ROS accumulates in middle lamella of peripheral cells of the LRP and of cell files overlying the LRP. The vascular localizations are omitted. Schematic representation is based on a TEM tissue section from Fig. 3. (B) Model of auxin- and RBOH-mediated ROS action during LRP emergence. For successful LR initiation and emergence, localized cell wall remodeling in front of LRP is required and relies on an orchestrated operation of several auxin response modules (Swarup et al., 2008). Here, we propose that ROS deposited into the cell walls by the activity of auxin-inducible RBOH enzymes facilitate LRP emergence by promoting cell wall remodeling.

Our findings that RBOH function contributes to LR emergence, which requires cell wall remodeling and accommodation, reveal a key role for RBOH in the control of apoplastic ROS production targeted to restricted spatial and temporal domains during organ outgrowth (Fig. 7). The restriction of RBOH expression to the peripheral cells of the LRP and to the cell files overlying it suggests that auxin signaling pathways control their expression pattern and potentially their activity and subsequent generation of ROS in the middle lamellae. We do not yet know whether induction of *RBOHE* expression in LRP-overlying cells is auxin regulated, perhaps in parallel with LAX3 in the LBD29/LAX3 signaling module (Porco et al., 2016). Hence, such precise ROS deposition suggests an intimate relationship between ROS and auxin-controlled changes in cell wall biomechanics during LRP emergence.

## MATERIALS AND METHODS

### Plant material and growth conditions

All *Arabidopsis* lines used in this study have been previously described: *AUX1:GUS* (Swarup et al., 2004), *LAX3:GUS* (Swarup et al., 2008), *pRBOH:nlsGFP:GUS* (Lee et al., 2013), *pCASP1:SHY2, pCASP1:shy2-22* (Vermeer et al., 2014), *iaa28-1* (Rogg et al., 2001), *slr* (Fukaki et al., 2002), *arf7 arf19* (Okushima et al., 2007), *aux1 lax3* (Swarup and Peret, 2012). The crosses were generated from the SAIL/SALK lines *rbohb* (SAIL_749_B11), *rbohc* (SALK_071801), *rbohd* (SALK_070610), *rbohe* (SALK_064850) and *rbohf* (SALK_059888) and were ordered from the Nottingham *Arabidopsis* Stock Centre.

The *GAL4* enhancer trap lines were ordered from the Nottingham *Arabidopsis* Stock Centre and crosses with a homozygous *UAS:RBOHD* were generated to produce transactivating lines. The *UAS:RBOHD* construct was generated by cloning the *RBOHD* cDNA into plasmid pDONR221 and next into the destination plasmid pKm34GW,0 simultaneously with the pEN-L4-UAS-R1 promoter and pEN-R2-NOS-L3 terminator sequences using a Gateway (Invitrogen) cloning approach. Transgenic plants were generated by a standard floral dip method.

In all experiments with *Arabidopsis*, seeds were sterilized with chlorine gas and stratified at 4°C for 2 days in water. After cold treatment, seeds were sown over solid half-strength Murashige and Skoog (MS) growth medium (per liter: 2.15 g MS salts, 0.1 g *myo*-inositol, 0.5 g MES, 10 g sucrose, 8 g plant tissue culture agar; pH 5.7 with KOH) (hereafter termed ‘medium’) and grown vertically under continuous light (110 µE m^–2^ s^–1^ photosynthetically active radiation, supplied by cool-white fluorescent tungsten tubes, Osram) for 4-5 days. The scans of the plates were taken with a V700 scanner (Epson) or 3200 dpi scanner (Medion). Seedlings were analyzed in detail with a BX53 microscope (Olympus) equipped with DS-Fi1 camera (Nikon). Figures were arranged in Photoshop CS3 (Adobe Systems) and the brightness was increased equally across samples, without further modifications. To characterize *GAL4* enhancer trap lines and transactivation lines, 5-day-old seedlings were imaged with an LSM5 (Axiovert, Zeiss) confocal microscope.

### Transmission electron microscopy

Cerium hydroxide precipitates indicate H_2_O_2_ localization. Five-day-old seedlings were gravistimulated by 90° to achieve synchronization of LR formation. After 22 h and 44 h, 2-mm fragments that were expected to contain early and late LRP were dissected under binoculars (*n*=50) and incubated for 1 h in 5 mM cerium chloride solution in 50 mM MOPS buffer [for 100 ml: 1.046 g of 3-(N-morpholino) propanesulfonic acid (MOPS; VWR Chemicals) in 90 ml of water and adjust the pH to 7.2 with 1.7 M Tris (VWR Chemicals)]. Tissue embedding and electron probe x-rays were performed as described (D'Haeze et al., 2003).

### qRT-PCR analysis

Col-0 seeds were sown on half-strength MS supplemented with 1% sucrose and grown for 7 days on a mesh. Seedlings were then transferred to 10 µM NAA for the indicated duration. RNA was extracted from dissected roots and 1 µg of RNA was used for cDNA synthesis and qRT-PCR analysis as described previously (Fernandez et al., 2013) with primer pairs as listed in Table S3. Data were analyzed with the ‘delta-delta method’ (Pfaffl, 2001), taking primer efficiency into consideration, and normalized with *UBIQUITIN 10* as reference transcript. The sample with the maximum value for each gene was chosen as the calibrator (set to 1), the results of two biological replicates were averaged and the expression values are given in arbitrary units relative to t0. *RBOHJ* and *RBOHH* primers only amplified in a few samples of the second replicate, consistent with the predicted expression pattern (not in roots), and are therefore not shown.

### LR phenotype analysis

Five-day-old *Arabidopsis* Col-0 and/or mutant seedlings were transferred on fresh media (control) or on media supplemented with various compounds, namely DPI (diphenyleneiodonium chloride, Sigma-Aldrich), DDC (diethyldithiocarbamate, Alfa Aesar/VWR Chemicals), paraquat (methyl viologen dichloride hydrate, Sigma-Aldrich), KI (potassium iodide, Applichem Lifescience). After 1 h, seedlings were gravistimulated by 90° to achieve synchronization of LR formation. After 20 h and 44 h, seedlings were pre-fixed in 0.4% formaldehyde (Sigma-Aldrich) in 50 mM phosphate buffer (VWR Chemicals) pH 7 at 4°C under a gentle vacuum for 30 min. Subsequently, 2.5 g of chloral hydrate (VWR Chemicals) was dissolved per 1 ml of 30% glycerol (Sigma-Aldrich) and seedlings were left overnight in a cleaning solution. LRP were observed with a BX53 dissecting microscope (Olympus) equipped with a DS-Fi1 (Nikon) camera and grouped according to developmental stages at 20 h and 44 h after the onset of gravistimulation.

### Histological staining

For DAB (diaminobenzidine tetrahydrochloride; Applichem Lifescience) and NBT (nitroblue tetrazolium chloride; Molekula/VWR Chemicals) staining in maize (B83 inbred line), the root segments were embedded in 6% agarose with 0.5% gelatine and 100-μm-thick sections were cut with a vibratome. Sections were immediately transferred for 1 h to NBT staining solution (0.1% NBT in 10 mM potassium phosphate buffer, pH 7.8) according to the methods of Kawai-Yamada et al. (2004) or for 2-3 h to DAB staining solution [1 mg/ml DAB, Tween 20 (0.05% v/v) and 10 mM Na_2_HPO_4_, pH>6.8] according to the methods of Daudi and O'Brien (2012). Upon signal development, sections were mounted with distilled water and immediately imaged with an AxioCam microscope (Zeiss).

For DCFH-DA (dichloro-dihydro-fluorescein diacetate; Sigma-Aldrich) staining in *Arabidopsis*, 5 dag seedlings were stained for 15 min in DCFH staining solution (50 μM DCFH-DA in 50 mM phosphate buffer) in darkness according to the methods of Shin et al. (2005). Seedlings were washed briefly in phosphate buffer alone before imaging by confocal microscopy using an LSM5 microscope (Axiovert, Zeiss).

### GUS staining

Seedlings were put overnight in 90% acetone, then transferred to a GUS-solution {1 mM X-Gluc, 0.5% (w/v) dimethylformamide (DMF), 0.5% (w/v) Triton X-100, 1 mM EDTA (pH 8), 0.5 mM potassium ferricyanide [K_3_Fe(CN)_6_], 0.5% potassium ferrocyanide [K_4_Fe(CN)_6_], 500 mM phosphate buffer (pH 7)} and incubated for 4 h at 37°C for GUS staining, and finally washed in 500 mM phosphate buffer (pH 7). For microscopic analysis, samples were cleared in chloral hydrate solution as described by Berleth and Jurgens (1993). Samples were analyzed by differential interference contrast microscopy with Primo Vert (Zeiss) equipped with Moticam 2300 (Motic).

### Treatment with auxin inhibitors

Seedlings (5 dgp) were transferred for 7 days to control growth media or to media supplemented with 10 μM 1-NOA (1-naphthoxyacetic acid; Alfa Aesar/VWR Chemicals), 10 μM TIBA (2,3,5-triiodobenzoic acid; Alfa Aesar/VWR Chemicals) and 1 μM NPA (N-1-naphthylphthalamic acid; Fluka/Sigma-Aldrich). The plates were scanned with a 3200 dpi scanner (Medion) and LR number was determined using a BX53 dissecting microscope (Olympus) equipped with DS-Fi1 (Nikon) camera.

### Microarray data retrieval, normalization and treatment

The following microarray hybridization files were retrieved from the Gene Expression Omnibus database: GEO series GSE3350 (GSM75508, GSM75509, GSM75512, GSM75513; Vanneste et al., 2005), series GSE42896 (GSM1053030, GSM1053031, GSM1053032, GSM1053036, GSM1053037, GSM1053038; De Rybel et al., 2012), series GSE41136 (GSM1009032, GSM1009033, GSM1009034, GSM1009029, GSM1009030, GSM1009031; Ng et al., 2013) and series GSE5530 (GSM128757, GSM128758, GSM128759, GSM128760, GSM128761, GSM128762; Davletova et al., 2005). Each dataset was been normalized independently with the robust multi-array average method and the differential analysis performed using the moderated *t*-test using the vignettes affy (Gautier et al., 2004) and limma (Smyth, 2005) within the R (www.r-project.org) bioconductor statistical package (www.bioconductor.org). Affymetrix probe sets to AGI ID assignment was performed using the affy_ATH1_array_elements-2010-12-20.txt file downloaded from TAIR (http://www.arabidopsis.org/download_files/Microarrays/Affymetrix/affy_ATH1_array_elements-2010-12-20.txt). A gene was considered as being differentially expressed if it fulfilled the following conditions: fold change ≥2 and *P*-value ≤0.05 in the two pairwise comparisons for the datasets related with NAA treatment, and at least in one of the two pairwise comparisons for the datasets related with H_2_O_2_ treatment. The number of probe sets that satisfied these criteria was 109, of which two were redundant, yielding a final list of 108 genes (Table S1). Gene ontologies were retrieved using Agrigo (http://bioinfo.cau.edu.cn/agriGO/) and TAIR (www.arabidopsis.org) databases.

### Statistical analyses

All data analyses were performed with R software package, v. 2.15. Different letters in figures indicate significant differences according to Tukey's HSD test after ANOVA unless stated otherwise.

## References

[DEV136465C1] Aranda A., Sequedo L., Tolosa L., Quintas G., Burello E., Castell J. V. and Gombau L. (2013). Dichloro-dihydro-fluorescein diacetate (DCFH-DA) assay: a quantitative method for oxidative stress assessment of nanoparticle-treated cells. *Toxicol. In vitro* 27, 954-963. 10.1016/j.tiv.2013.01.016 23357416

[DEV136465C2] Benkova E., Michniewicz M., Sauer M., Teichmann T., Seifertova D., Jurgens G. and Friml J. (2003). Local, efflux-dependent auxin gradients as a common module for plant organ formation. *Cell* 115, 591-602. 10.1016/S0092-8674(03)00924-3 14651850

[DEV136465C3] Berleth T. and Jurgens G. (1993). The role of monopteros in organizing the basal body region of the Arabidoposis embryo. *Development* 118, 575-587.

[DEV136465C4] Bibikova T. N., Jacob T., Dahse I. and Gilroy S. (1998). Localized changes in apoplastic and cytoplasmic pH are associated with root hair development in Arabidopsis thaliana. *Development* 125, 2925-2934. 9655814 10.1242/dev.125.15.2925

[DEV136465C5] Boisson-Dernier A., Lituiev D. S., Nestorova A., Franck C. M., Thirugnanarajah S. and Grossniklaus U. (2013). ANXUR receptor-like kinases coordinate cell wall integrity with growth at the pollen tube tip via NADPH oxidases. *PLoS Biol.* 11, e1001719 10.1371/journal.pbio.1001719 24302886 PMC3841104

[DEV136465C6] Carol R. J., Takeda S., Linstead P., Durrant M. C., Kakesova H., Derbyshire P., Drea S., Zarsky V. and Dolan L. (2005). A RhoGDP dissociation inhibitor spatially regulates growth in root hair cells. *Nature* 438, 1013-1016. 10.1038/nature04198 16355224

[DEV136465C7] Casimiro I., Marchant A., Bhalerao R. P., Beeckman T., Dhooge S., Swarup R., Graham N., Inze D., Sandberg G., Casero P. J. et al. (2001). Auxin transport promotes Arabidopsis lateral root initiation. *Plant Cell* 13, 843-852. 10.1105/tpc.13.4.843 11283340 PMC135543

[DEV136465C8] Caverzan A., Passaia G., Rosa S. B., Ribeiro C. W., Lazzarotto F. and Margis-Pinheiro M. (2012). Plant responses to stresses: role of ascorbate peroxidase in the antioxidant protection. *Genet. Mol. Biol.* 35, 1011-1019. 10.1590/S1415-47572012000600016 23412747 PMC3571416

[DEV136465C9] Correa-Aragunde N., Foresi N., Delledonne M. and Lamattina L. (2013). Auxin induces redox regulation of ascorbate peroxidase 1 activity by S-nitrosylation/denitrosylation balance resulting in changes of root growth pattern in Arabidopsis. *J. Exp. Bot.* 64, 3339-3349. 10.1093/jxb/ert172 23918967

[DEV136465C10] Daudi A. and O'Brien J. A. (2012). Detection of hydrogen peroxide by DAB staining in Arabidopsis leaves. *Bio Protoc.* 2, e263. PMC4932902 27390754

[DEV136465C11] Davletova S., Schlauch K., Coutu J. and Mittler R. (2005). The zinc-finger protein Zat12 plays a central role in reactive oxygen and abiotic stress signaling in Arabidopsis. *Plant Physiol.* 139, 847-856. 10.1104/pp.105.068254 16183833 PMC1256000

[DEV136465C12] De Rybel B., Audenaert D., Xuan W., Overvoorde P., Strader L. C., Kepinski S., Hoye R., Brisbois R., Parizot B., Vanneste S. et al. (2012). A role for the root cap in root branching revealed by the non-auxin probe naxillin. *Nat. Chem. Biol.* 8, 798-805. 10.1038/nchembio.1044 22885787 PMC3735367

[DEV136465C13] De Smet I. (2011). Lateral root initiation: one step at a time. *New Phytol.* 193, 867-873. 10.1111/j.1469-8137.2011.03996.x 22403823

[DEV136465C14] De Smet I., Zhang H., Inze D. and Beeckman T. (2006). A novel role for abscisic acid emerges from underground. *Trends Plant Sci.* 11, 434-439. 10.1016/j.tplants.2006.07.003 16890475

[DEV136465C15] De Smet I., Tetsumura T., De Rybel B., Frey N. F. D., Laplaze L., Casimiro I., Swarup R., Naudts M., Vanneste S., Audenaert D. et al. (2007). Auxin-dependent regulation of lateral root positioning in the basal meristem of Arabidopsis. *Development* 134, 681-690. 10.1242/dev.02753 17215297

[DEV136465C16] D'Haeze W., De Rycke R., Mathis R., Goormachtig S., Pagnotta S., Verplancke C., Capoen W. and Holsters M. (2003). Reactive oxygen species and ethylene play a positive role in lateral root base nodulation of a semiaquatic legume. *Proc. Natl. Acad. Sci. USA* 100, 11789-11794. 10.1073/pnas.1333899100 12975522 PMC208836

[DEV136465C17] Duan L., Dietrich D., Ng C. H., Chan P. M. Y., Bhalerao R., Bennett M. J. and Dinneny J. R. (2013). Endodermal ABA signaling promotes lateral root quiescence during salt stress in Arabidopsis seedlings. *Plant Cell* 25, 324-341. 10.1105/tpc.112.107227 23341337 PMC3584545

[DEV136465C18] Dubrovsky J. G., Gambetta G. A., Hernandez-Barrera A., Shishkova S. and Gonzalez I. (2006). Lateral root initiation in Arabidopsis: developmental window, spatial patterning, density and predictability. *Ann. Bot.* 97, 903-915. 10.1093/aob/mcj604 16390845 PMC2803408

[DEV136465C19] Fernandez A., Drozdzecki A., Hoogewijs K., Nguyen A., Beeckman T., Madder A. and Hilson P. (2013). Transcriptional and functional classification of the GOLVEN/ROOT GROWTH FACTOR/CLE-like signaling peptides reveals their role in lateral root and hair formation. *Plant Physiol.* 161, 954-970. 10.1104/pp.112.206029 23370719 PMC3561032

[DEV136465C20] Foreman J., Demidchik V., Bothwell J. H. F., Mylona P., Miedema H., Torres M. A., Linstead P., Costa S., Brownlee C., Jones J. D. G. et al. (2003). Reactive oxygen species produced by NADPH oxidase regulate plant cell growth. *Nature* 422, 442-446. 10.1038/nature01485 12660786

[DEV136465C21] Fukaki H., Tameda S., Masuda H. and Tasaka M. (2002). Lateral root formation is blocked by a gain-of-function mutation in the SOLITARY-ROOT/IAA14 gene of Arabidopsis. *Plant J.* 29, 153-168. 10.1046/j.0960-7412.2001.01201.x 11862947

[DEV136465C122] Gautier L., Cope L., Bolstad B. M. and Irizarry R. A. (2004). affy analysis of Affymetrix GeneChip data at the probe level. *Bioinformatics* 20, 307-315. 10.1093/bioinformatics/btg405 14960456

[DEV136465C22] Geldner N., Richter S., Vieten A., Marquardt S., Torres-Ruiz R. A., Mayer U. and Jurgens G. (2004). Partial loss-of-function alleles reveal a role for GNOM in auxin transport-related, post-embryonic development of Arabidopsis. *Development* 131, 389-400. 10.1242/dev.00926 14681187

[DEV136465C23] Gjetting S. K., Ytting C. K., Schulz A. and Fuglsang A. T. (2012). Live imaging of intra- and extracellular pH in plants using pHusion, a novel genetically encoded biosensor. *J. Exp. Bot.* 63, 3207-3218. 10.1093/jxb/ers040 22407646 PMC3350929

[DEV136465C24] Goh T., Kasahara H., Mimura T., Kamiya Y. and Fukaki H. (2012). Multiple AUX/IAA-ARF modules regulate lateral root formation: the role of Arabidopsis SHY2/IAA3-mediated auxin signalling. *Philos. Trans. R. Soc. Lond. B Biol. Sci.* 367, 1461-1468. 10.1098/rstb.2011.0232 22527388 PMC3321683

[DEV136465C25] Gonzalez-Carranza Z. H., Elliott K. A. and Roberts J. A. (2007). Expression of polygalacturonases and evidence to support their role during cell separation processes in Arabidopsis thaliana. *J. Exp. Bot.* 58, 3719-3730. 10.1093/jxb/erm222 17928369

[DEV136465C26] Himanen K., Boucheron E., Vanneste S., de Almeida Engler J., Inze D. and Beeckman T. (2002). Auxin-mediated cell cycle activation during early lateral root initiation. *Plant Cell* 14, 2339-2351. 10.1105/tpc.004960 12368490 PMC151221

[DEV136465C27] Hosmani P. S., Kamiya T., Danku J., Naseer S., Geldner N., Guerinot M. L. and Salt D. E. (2013). Dirigent domain-containing protein is part of the machinery required for formation of the lignin-based Casparian strip in the root. *Proc. Natl. Acad. Sci. USA* 110, 14498-14503. 10.1073/pnas.1308412110 23940370 PMC3761638

[DEV136465C28] Ishibashi Y., Tawaratsumida T., Kondo K., Kasa S., Sakamoto M., Aoki N., Zheng S.-H., Yuasa T. and Iwaya-Inoue M. (2012). Reactive oxygen species are involved in gibberellin/abscisic acid signaling in barley aleurone cells. *Plant Physiol.* 158, 1705-1714. 10.1104/pp.111.192740 22291200 PMC3320179

[DEV136465C29] Ivanchenko M. G., den Os D., Monshausen G. B., Dubrovsky J. G., Bednarova A. and Krishnan N. (2013). Auxin increases the hydrogen peroxide (H2O2) concentration in tomato (Solanum lycopersicum) root tips while inhibiting root growth. *Ann. Bot.* 112, 1107-1116. 10.1093/aob/mct181 23965615 PMC3783245

[DEV136465C30] Jansen L., Parizot B. and Beeckman T. (2013). Inducible system for lateral roots in Arabidopsis thaliana and maize. *Methods Mol. Biol.* 959, 149-158. 10.1007/978-1-62703-221-6_9 23299673

[DEV136465C31] Joo J. H., Bae Y. S. and Lee J. S. (2001). Role of auxin-induced reactive oxygen species in root gravitropism. *Plant Physiol.* 126, 1055-1060. 10.1104/pp.126.3.1055 11457956 PMC116462

[DEV136465C32] Kawai-Yamada M., Ohori Y. and Uchimiya H. (2004). Dissection of Arabidopsis Bax inhibitor-1 suppressing Bax-, hydrogen peroxide-, and salicylic acid-induced cell death. *Plant Cell* 16, 21-32. 10.1105/tpc.014613 14671021 PMC301392

[DEV136465C33] Kumpf R. P., Shi C.-L., Larrieu A., Sto I. M., Butenko M. A., Peret B., Riiser E. S., Bennett M. J. and Aalen R. B. (2013). Floral organ abscission peptide IDA and its HAE/HSL2 receptors control cell separation during lateral root emergence. *Proc. Natl. Acad. Sci. USA* 110, 5235-5240. 10.1073/pnas.1210835110 23479623 PMC3612645

[DEV136465C34] Kwak J. M., Mori I. C., Pei Z.-M., Leonhardt N., Torres M. A., Dangl J. L., Bloom R. E., Bodde S., Jones J. D. G. and Schroeder J. I. (2003). NADPH oxidase AtrbohD and AtrbohF genes function in ROS-dependent ABA signaling in Arabidopsis. *EMBO J.* 22, 2623-2633. 10.1093/emboj/cdg277 12773379 PMC156772

[DEV136465C35] Lavenus J., Goh T., Roberts I., Guyomarc'h S., Lucas M., De Smet I., Fukaki H., Beeckman T., Bennett M. and Laplaze L. (2013). Lateral root development in Arabidopsis: fifty shades of auxin. *Trends Plant Sci.* 18, 450-458. 10.1016/j.tplants.2013.04.006 23701908

[DEV136465C36] Lee Y., Rubio M. C., Alassimone J. and Geldner N. (2013). A mechanism for localized lignin deposition in the endodermis. *Cell* 153, 402-412. 10.1016/j.cell.2013.02.045 23541512

[DEV136465C37] Lewis D. R., Olex A. L., Lundy S. R., Turkett W. H., Fetrow J. S. and Muday G. K. (2013). A kinetic analysis of the auxin transcriptome reveals cell wall remodeling proteins that modulate lateral root development in Arabidopsis. *Plant Cell* 25, 3329-3346. 10.1105/tpc.113.114868 24045021 PMC3809535

[DEV136465C38] Li J. and Jia H. (2013). Hydrogen peroxide is involved in cGMP modulating the lateral root development of Arabidopsis thaliana. *Plant Signal. Behav.* 8, e25052 10.4161/psb.25052 23733053 PMC3999063

[DEV136465C39] Liao W.-B., Zhang M.-L., Huang G.-B. and Yu J.-H. (2012). Ca2+ and CaM are involved in NO- and H2O2-induced adventitious root development in marigold. *J. Plant Growth Regul.* 31, 253-264. 10.1007/s00344-011-9235-7

[DEV136465C40] Ma F., Wang L., Li J., Samma M. K., Xie Y., Wang R., Wang J., Zhang J. and Shen W. (2014). Interaction between HY1 and H2O2 in auxin-induced lateral root formation in Arabidopsis. *Plant Mol. Biol.* 85, 49-61. 10.1007/s11103-013-0168-3 24366686

[DEV136465C41] Malamy J. E. and Benfey P. N. (1997). Organization and cell differentiation in lateral roots of Arabidopsis thaliana. *Development* 124, 33-44. 9006065 10.1242/dev.124.1.33

[DEV136465C42] Manzano C., Pallero-Baena M., Casimiro I., De Rybel B., Orman-Ligeza B., Van Isterdael G., Beeckman T., Draye X., Casero P. and Del Pozo J. C. (2014). The emerging role of reactive oxygen species signaling during lateral root development. *Plant Physiol.* 165, 1105-1119. 10.1104/pp.114.238873 24879433 PMC4081325

[DEV136465C43] Marchant A., Bhalerao R., Casimiro I., Eklof J., Casero P. J., Bennett M. and Sandberg G. (2002). AUX1 promotes lateral root formation by facilitating indole-3-acetic acid distribution between sink and source tissues in the Arabidopsis seedling. *Plant Cell* 14, 589-597. 10.1105/tpc.010354 11910006 PMC150581

[DEV136465C44] Marhavy P., Vanstraelen M., De Rybel B., Zhaojun D., Bennett M. J., Beeckman T. and Benkova E. (2013). Auxin reflux between the endodermis and pericycle promotes lateral root initiation. *EMBO J.* 32, 149-158. 10.1038/emboj.2012.303 23178590 PMC3545298

[DEV136465C45] Mersmann S., Bourdais G., Rietz S. and Robatzek S. (2010). Ethylene signaling regulates accumulation of the FLS2 receptor and is required for the oxidative burst contributing to plant immunity. *Plant Physiol.* 154, 391-400. 10.1104/pp.110.154567 20592040 PMC2938167

[DEV136465C46] Monshausen G. B., Bibikova T. N., Messerli M. A., Shi C. and Gilroy S. (2007). Oscillations in extracellular pH and reactive oxygen species modulate tip growth of Arabidopsis root hairs. *Proc. Natl. Acad. Sci. USA* 104, 20996-21001. 10.1073/pnas.0708586104 18079291 PMC2409255

[DEV136465C47] Mori I. C., Pinontoan R., Kawano T. and Muto S. (2001). Involvement of superoxide generation in salicylic acid-induced stomatal closure in Vicia faba. *Plant Cell Physiol.* 42, 1383-1388. 10.1093/pcp/pce176 11773531

[DEV136465C48] Muller K., Carstens A. C., Linkies A., Torres M. A. and Leubner-Metzger G. (2009). The NADPH-oxidase AtrbohB plays a role in Arabidopsis seed after-ripening. *New Phytol.* 184, 885-897. 10.1111/j.1469-8137.2009.03005.x 19761445

[DEV136465C49] Neuteboom L. W., Veth-Tello L. M., Clijdesdale O. R., Hooykaas P. J. J. and van der Zaal B. J. (1999). A novel subtilisin-like protease gene from Arabidopsis thaliana is expressed at sites of lateral root emergence. *DNA Res.* 6, 13-19. 10.1093/dnares/6.1.13 10231025

[DEV136465C50] Ng S., Ivanova A., Duncan O., Law S. R., Van Aken O., De Clercq I., Wang Y., Carrie C., Xu L., Kmiec B. et al. (2013). A membrane-bound NAC transcription factor, ANAC017, mediates mitochondrial retrograde signaling in Arabidopsis. *Plant Cell* 25, 3450-3471. 10.1105/tpc.113.113985 24045017 PMC3809543

[DEV136465C51] Okushima Y., Fukaki H., Onoda M., Theologis A. and Tasaka M. (2007). ARF7 and ARF19 regulate lateral root formation via direct activation of LBD/ASL genes in Arabidopsis. *Plant Cell* 19, 118-130. 10.1105/tpc.106.047761 17259263 PMC1820965

[DEV136465C52] Passaia G., Spagnolo Fonini L., Caverzan A., Jardim-Messeder D., Christoff A. P., Gaeta M. L., de Araujo Mariath J. E., Margis R. and Margis-Pinheiro M. (2013). The mitochondrial glutathione peroxidase GPX3 is essential for H2O2 homeostasis and root and shoot development in rice. *Plant Sci.* 208, 93-101. 10.1016/j.plantsci.2013.03.017 23683934

[DEV136465C53] Peret B., Li G., Zhao J., Band L. R., Voss U., Postaire O., Luu D.-T., Da Ines O., Casimiro I., Lucas M. et al. (2012a). Auxin regulates aquaporin function to facilitate lateral root emergence. *Nat. Cell Biol.* 14, 991-998. 10.1038/ncb2573 22983115

[DEV136465C54] Peret B., Swarup K., Ferguson A., Seth M., Yang Y., Dhondt S., James N., Casimiro I., Perry P., Syed A. et al. (2012b). AUX/LAX genes encode a family of auxin influx transporters that perform distinct functions during Arabidopsis development. *Plant Cell* 24, 2874-2885. 10.1105/tpc.112.097766 22773749 PMC3426120

[DEV136465C55] Peret B., Middleton A. M., French A. P., Larrieu A., Bishopp A., Njo M., Wells D. M., Porco S., Mellor N., Band L. R. et al. (2013). Sequential induction of auxin efflux and influx carriers regulates lateral root emergence. *Mol. Syst. Biol.* 9, 699 10.1038/msb.2013.43 24150423 PMC3817398

[DEV136465C56] Pfaffl M. W. (2001). A new mathematical model for relative quantification in real-time RT-PCR. *Nucleic Acids Res.* 29, e45 10.1093/nar/29.9.e45 11328886 PMC55695

[DEV136465C57] Porco S., Larrieu A., Du Y., Gaudinier A., Goh T., Swarup K., Swarup R., Kuempers B., Bishopp A., Lavenus J. et al. (2016). Lateral root emergence in *Arabidopsis* is dependent on transcription factor LBD29 regulation of auxin influx carrier *LAX3*. *Development* 143, 3340-3349. 27578783 10.1242/dev.136283

[DEV136465C58] Rogg L. E., Lasswell J. and Bartel B. (2001). A gain-of-function mutation in IAA28 suppresses lateral root development. *Plant Cell* 13, 465-480. 10.1105/tpc.13.3.465 11251090 PMC135515

[DEV136465C59] Ros Barcelo A. (2005). Xylem parenchyma cells deliver the H2O2 necessary for lignification in differentiating xylem vessels. *Planta* 220, 747-756. 10.1007/s00425-004-1394-3 15747145

[DEV136465C60] Roycewicz P. S. and Malamy J. E. (2014). Cell wall properties play an important role in the emergence of lateral root primordia from the parent root. *J. Exp. Bot.* 65, 2057-2069. 10.1093/jxb/eru056 24619997 PMC3991740

[DEV136465C61] Sagi M. and Fluhr R. (2006). Production of reactive oxygen species by plant NADPH oxidases. *Plant Physiol.* 141, 336-340. 10.1104/pp.106.078089 16760484 PMC1475462

[DEV136465C62] Shapiguzov A., Vainonen J. P., Wrzaczek M. and Kangasjarvi J. (2012). ROS-talk - how the apoplast, the chloroplast, and the nucleus get the message through. *Front. Plant Sci.* 3, 292 10.3389/fpls.2012.00292 23293644 PMC3530830

[DEV136465C63] Shin, R., Berg R. H. and Schachtman D. P. (2005). Reactive oxygen species and root hairs in Arabidopsis root response to nitrogen, phosphorus and potassium deficiency. *Plant Cell Physiol.* 46, 1350-1357. 10.1093/pcp/pci145 15946982

[DEV136465C164] Smyth G., Michaud J. and Scott H. (2005). Use of within-array replicate spots for assessing differential expression in microarray experiments. *Bioinformatics* 21, 2067-2075 10.1093/bioinformatics/bti270 15657102

[DEV136465C64] Swarup R. and Peret B. (2012). AUX/LAX family of auxin influx carriers-an overview. *Front. Plant Sci.* 3, 225 10.3389/fpls.2012.00225 23087694 PMC3475149

[DEV136465C65] Swarup R., Kargul J., Marchant A., Zadik D., Rahman A., Mills R., Yemm A., May S., Williams L., Millner P. et al. (2004). Structure-function analysis of the presumptive Arabidopsis auxin permease AUX1. *Plant Cell* 16, 3069-3083. 10.1105/tpc.104.024737 15486104 PMC527199

[DEV136465C66] Swarup K., Benkova E., Swarup R., Casimiro I., Peret B., Yang Y., Parry G., Nielsen E., De Smet I., Vanneste S. et al. (2008). The auxin influx carrier LAX3 promotes lateral root emergence. *Nat. Cell Biol.* 10, 946-954. 10.1038/ncb1754 18622388

[DEV136465C67] Tian Q. and Reed J. W. (1999). Control of auxin-regulated root development by the Arabidopsis thaliana SHY2/IAA3 gene. *Development* 126, 711-721. 9895319 10.1242/dev.126.4.711

[DEV136465C68] Torres M. A., Dangl J. L. and Jones J. D. G. (2002). Arabidopsis gp91phox homologues AtrbohD and AtrbohF are required for accumulation of reactive oxygen intermediates in the plant defense response. *Proc. Natl. Acad. Sci. USA* 99, 517-522. 10.1073/pnas.012452499 11756663 PMC117592

[DEV136465C69] Van Norman J. M., Zhang J., Cazzonelli C. I., Pogson B. J., Harrison P. J., Bugg T. D. H., Chan K. X., Thompson A. J. and Benfey P. N. (2014). Periodic root branching in Arabidopsis requires synthesis of an uncharacterized carotenoid derivative. *Proc. Natl. Acad. Sci. USA* 111, E1300-E1309. 10.1073/pnas.1403016111 24639533 PMC3977299

[DEV136465C70] Vanneste S., De Rybel B., Beemster G. T., Ljung K., De Smet I., Van Isterdael G., Naudts M., Iida R., Gruissem W., Tasaka M. et al. (2005). Cell cycle progression in the pericycle is not sufficient for SOLITARY ROOT/IAA14-mediated lateral root initiation in Arabidopsis thaliana. *Plant Cell* 17, 3035-3050. 10.1105/tpc.105.035493 16243906 PMC1276028

[DEV136465C71] Vermeer J. E. M., von Wangenheim D., Barberon M., Lee Y., Stelzer E. H. K., Maizel A. and Geldner N. (2014). A spatial accommodation by neighboring cells is required for organ initiation in Arabidopsis. *Science* 343, 178-183. 10.1126/science.1245871 24408432

